# Morphology, Carbohydrate Distribution, Gene Expression, and Enzymatic Activities Related to Cell Wall Hydrolysis in Four Barley Varieties during Simulated Malting

**DOI:** 10.3389/fpls.2017.01872

**Published:** 2017-10-30

**Authors:** Natalie S. Betts, Laura G. Wilkinson, Shi F. Khor, Neil J. Shirley, Finn Lok, Birgitte Skadhauge, Rachel A. Burton, Geoffrey B. Fincher, Helen M. Collins

**Affiliations:** ^1^Australian Research Council Centre of Excellence in Plant Cell Walls and School of Agriculture, Food and Wine, University of Adelaide, Waite, Glen Osmond, SA, Australia; ^2^Carlsberg Research Laboratory, Copenhagen, Denmark

**Keywords:** malting, barley, cell wall, *Hordeum vulgare*, enzymes, germination, grain morphology

## Abstract

Many biological processes, such as cell wall hydrolysis and the mobilisation of nutrient reserves from the starchy endosperm, require stringent regulation to successfully malt barley (*Hordeum vulgare*) grain in an industrial context. Much of the accumulated knowledge defining these events has been collected from individual, unrelated experiments, and data have often been extrapolated from Petri dish germination, rather than malting, experiments. Here, we present comprehensive morphological, biochemical, and transcript data from a simulated malt batch of the three elite malting cultivars Admiral, Navigator, and Flagship, and the feed cultivar Keel. Activities of lytic enzymes implicated in cell wall and starch depolymerisation in germinated grain have been measured, and transcript data for published cell wall hydrolytic genes have been provided. It was notable that Flagship and Keel exhibited generally similar patterns of enzyme and transcript expression, but exhibited a few key differences that may partially explain Flagship's superior malting qualities. Admiral and Navigator also showed matching expression patterns for these genes and enzymes, but the patterns differed from those of Flagship and Keel, despite Admiral and Navigator having Keel as a common ancestor. Overall (1,3;1,4)-β-glucanase activity differed between cultivars, with lower enzyme levels and concomitantly higher amounts of (1,3;1,4)-β-glucan in the feed variety, Keel, at the end of malting. Transcript levels of the gene encoding (1,3;1,4)-β-glucanase isoenzyme EI were almost three times higher than those encoding isoenzyme EII, suggesting a previously unrecognised importance for isoenzyme EI during malting. Careful morphological examination showed that scutellum epithelial cells in mature dry grain are elongated but expand no further as malting progresses, in contrast to equivalent cells in other cereals, perhaps demonstrating a morphological change in this critical organ over generations of breeding selection. Fluorescent immuno-histochemical labelling revealed the presence of pectin in the nucellus and, for the first time, significant amounts of callose throughout the starchy endosperm of mature grain.

## Introduction

The barley grain consists of embryonic, endosperm and outer protective tissues that play different roles throughout development and germination. The outer maternal tissues consist of the hull (husk), pericarp, testa, and nucellus, which protect the grain from mechanical damage and pathogen attack during harvest and germination (Freeman and Palmer, 1984). The husk consists of the lemma on the dorsal side and the palea on the ventral, or furrowed, side of the grain, and accounts for about 10% of grain weight (Fox, 2009). In hulled varieties, the palea and lemma become fixed to the pericarp of mature grain (Duffus and Cochrane, 1993), in contrast with hull-less varieties and some other cereals including wheat and rice, in which the palea and lemma are loose and are dislodged and removed during threshing. The pericarp is the residual fruit wall that develops from the ovary wall (Duffus and Cochrane, 1992), and lies just below the husk. During grain development, photosynthesis occurs in cells of the pericarp but these cells die during grain maturation. The seed coat, or testa, forms from the inner integument and covers the whole grain except for a small region at the embryo end, where it is penetrated by the micropyle, through which water enters the grain (Duffus and Cochrane, 1992). The nucellus, which plays a crucial role in feeding the embryo during early development, persists only as a crushed epidermal layer under the testa (Brown and Morris, 1890; Bacic and Stone, 1981b).

The embryonic tissues consist of the axis and the scutellum. The axis contains the root and shoot initials, which are surrounded by the coleorhiza and coleoptile, respectively. The scutellum is a modified cotyledon that lies adjacent to the starchy endosperm, and mediates the secretion of hormones and lytic enzymes to the aleurone and starchy endosperm, and the subsequent transport of nutrients back to the growing embryo (Walker-Smith and Payne, 1984). The single layer of cells adjacent to the endosperm is called the scutellar epithelium and contains cells that are elongated perpendicular to the interface with the endosperm (Brown and Morris, 1890; O'Brien, 1942, 1951; Smart and O'Brien, 1979).

The endosperm consists of the starchy endosperm and the aleurone, which differentiates into a discrete tissue around the starchy endosperm during grain development (Wilson et al., 2006). The starchy endosperm is high in starch and protein reserves but also contains cell wall material and residual nucleic acids (Fincher, 1989). Aleurone cells respond to gibberellic acid during germination to produce hydrolytic enzymes that are secreted into the starchy endosperm for the mobilisation of grain reserves (Fincher, 1989).

Biologically, germination begins with water uptake (imbibition) and is complete when the embryonic axis emerges from the seed (Bewley and Black, 1994). Malting is a specialised, regulated germination process that prepares barley grain for efficient brewing, by activating enzymes and initiating the mobilisation of starch and protein reserves. Grain is immersed in water (steeped) for up to 24 h, allowed to germinate under controlled conditions, and finally kiln-dried to halt seedling growth. Here, we use industry terminology to refer to the stages in the malting process as steeping, germination, and kilning (Bewley and Black, 1994).

Enzymes active during malting may be categorised into three major functional groups: starch hydrolases that collectively contribute to the diastatic power of malt; cell wall hydrolases that are responsible for the degradation of cell wall polysaccharides; and proteolytic enzymes, which partially mobilise storage proteins of the starchy endosperm. In the present study, we have focused on previously described genes and enzymes that mediate the hydrolysis of cell wall polysaccharides during barley germination. While we have included activity data for enzymes involved in starch hydrolysis, we have not included transcript analyses for genes encoding enzymes involved in starch or protein hydrolysis, because the barley genome sequence (Mascher et al., 2017) has revealed that important families of these genes are much larger and more complex than previously thought (NS Betts, HM Collins and GB Fincher, unpublished data).

Breakdown of starch, the major carbohydrate nutrient of the endosperm, is achieved by the coordinated activity of enzymes from four major families, namely α-amylase, β-amylase, starch debranching enzymes (including limit dextrinase), and α-glucosidase. Amylose and amylopectin are internally cleaved by the endo-acting α-amylase, maltose is liberated from the non-reducing end of starch molecules by the exo-hydrolase β-amylase, limit dextrinase hydrolyses the (1,6)-α-linkages that form the branch points of amylopectin, and glucose is released from the resulting oligosaccharides by α-glucosidase. α-Amylase and α-glucosidase are known to be transcribed and translated *de novo* during germination (MacGregor and Lenoir, 1987), but β-amylase and limit dextrinase are transcribed and translated during grain development, and held inactive in protein complexes until germination commences (Hara-Nishimura et al., 1986; Guerin et al., 1992; Grime and Briggs, 1996).

Cell walls of the starchy endosperm are composed of approximately 70% (1,3;1,4)-β-glucan and 20% arabinoxylan (Fincher, 1989), and may account for up to 20% of the carbohydrate released from the starchy endosperm (Briggs, 1973). Aleurone cell walls of barley consist of approximately 20% (1,3;1,4)-β-glucan and 75% arabinoxylan (Bacic and Stone, 1981a; Fincher and Stone, 1993). Also present in starchy endosperm and aleurone cell walls are small amounts of cellulose and glucomannans, together with extracellular (1,3)-β-glucan (callose) (Fincher, 1989). The presence of callose has been reported to be transient during endosperm development and has been found in the mature starchy endosperm in small deposits adjacent to the aleurone layer (Fulcher et al., 1977; Wilson et al., 2012).

The (1,3;1,4)-β-glucan of the barley endosperm is completely hydrolysed to glucose by the concerted action of (1,3;1,4)-β-glucan endohydrolases, exo-acting β-glucan glucohydrolases, and β-glucosidases. The depolymerisation of arabinoxylan molecules is more complex. Arabinosyl residues are removed by the action of the arabinoxylan arabinofuranohydrolase (AXAH) enzymes (Lee et al., 2001; Simpson et al., 2003; Laidlaw et al., 2012), while the xylan backbone is degraded by (1,4)-β-endoxylanase isoenzymes (Banik et al., 1997; Sungurtas et al., 2004). β-Xylosidases hydrolyse xylan oligosaccharides while the α-l-arabinofuranosidase Ara1 is a bifunctional enzyme with both α-l-arabinofuranosidase and β-d-xylosidase activity (Lee et al., 2001; Laidlaw et al., 2012).

In this study, we have malted barley under conditions that closely simulate commercial malting processes, rather than using traditional Petri dish-like germination experimental systems. Conditions in a commercial malting plant result in lower oxygen, moisture, and often lack the free flow of carbon dioxide available to grains germinating naturally. Also, variations in temperature and anoxia result in little root development (Kleinwächter et al., 2012), which can lead to changes at a molecular level and hence incorrect conclusions when extrapolated to the harsh conditions of a commercial malting plant.

We have monitored morphological, biochemical, and transcriptional changes in three elite Australian malting cultivars and one feed cultivar and compared expression patterns of selected genes and enzymes. While some of these enzyme activity and gene transcript profile data have been reported previously, this is the first time that such a large number of enzymes and genes has been assessed in the same malted grains, allowing direct comparisons between varieties. We have also used both staining and immuno-histochemical techniques to link morphological and compositional changes with the activities of cell wall and starch hydrolytic enzymes during the small-scale simulated malt.

## Materials and methods

### Germination conditions

*Hordeum vulgare* cultivars Admiral, Flagship, Keel, and Navigator were grown at Charlick SA, in 2013 by the Barley Breeding Program of the University of Adelaide. The varieties Admiral and Navigator were both released in 2011 and have the feed variety Keel in their pedigree. Flagship and Keel were released in 2006 and 1999, respectively. Details of their origin and breeding can be found in the Australian PBR database (https://www.ipaustralia.gov.au/). The protein contents of grain samples were 8.7–9.4% w/w.

To simulate a malting process, the grain was germinated in the dark at 16°C using a regime of 6 h steep, 10 h air rest, 2 h steep, and 96 h germination. Throughout germination, grain weight was monitored to maintain moisture content at 40–44%. Grain was collected at 0, 3, 6, 16, and 18 h of the steeping phase, and every 24 h during the germination phase (Table 1). Grains were either fixed for microscopy or frozen in liquid nitrogen and stored at −80°C prior to analysis.

**Table 1:** The malting regime used to prepare grain.

**Sample**	**Hours after imbibition (hai)**	**Steeping**			**Germination**			
		**6 h wet**	**10 h air rest**	**2 h wet**	**24 h**	**48 h**	**72 h**	**96 h**
1	0							
2	3	✓(12)						
3	6	✓						
4	16	✓	✓					
5	18	✓	✓	✓				
6	42	✓	✓	✓				
7	66	✓	✓	✓	✓	✓		
8	90	✓	✓	✓	✓	✓	✓	
9	114	✓	✓	✓	✓	✓	✓	✓

### Fixing and embedding grain sections

For microscopy, grain was dissected either transversely or longitudinally. The embryo-containing segments were fixed in 0.25% glutaraldehyde, 4% paraformaldehyde, 4% sucrose in PBS, dehydrated in an ethanol series, and embedded in LR White Resin (ProSciTech Pty. Ltd., Australia) according to Burton et al. (2011). Sections (1 μm) were prepared with an ultramicrotome using a diamond knife, and dried onto glass microscope slides. Sections for morphology analysis were stained with toluidine blue O (Sigma-Aldrich) and photographed on a Nikon Ni-E microscope or a Carl Zeiss M2 AxioImager microscope. Negative controls are presented in Figure S1.

### Immuno-histochemical microscopy

Fluorescent immuno-histochemistry microscopy was performed as described by Burton et al. (2011), employing Calcofluor White Stain (Sigma F3543) and primary monoclonal antibodies (diluted 1/50). The antibodies used were BG1 murine monoclonal antibody to (1,3;1,4)-β-glucan (Meikle et al., 1994) (Biosupplies Australia, Parkville), LM11 antibody to xylan/arabinoxylan (McCartney et al., 2005), (1,3)-β-glucan murine monoclonal antibody (Meikle et al., 1991), LM19 antibody to homogalacturonan (Verhertbruggen et al., 2009), LM20 antibody to methyl-esterified homoglacturonan (Verhertbruggen et al., 2009), and CBM3a cellulose binding module (McCartney et al., 2004; Tan et al., 2015). Alexa Fluor® 488 goat anti-mouse IgG (H+L) was used as the secondary antibody to BG1 and (1,3)-β-glucan, Alexa Fluor® 555 goat anti-mouse IgG was used with LM11 and Alexa Fluor® 550 goat anti-rat IgM for LM19 and LM20 (all diluted 1:200, Invitrogen, Australia). For CBM3a, a two stage secondary antibody phase was employed using a mouse anti-histidine monoclonal antibody (1:100 dilution, Sigma-Aldrich) followed by Alexa Fluor® 488 goat anti-mouse IgG (1:100 dilution, Invitrogen) as described in Tan et al. (2015). Fluorescence was observed using a Carl Zeiss M2 AxioImager microscope with an AxioCam Mrm camera, and subsequent image processing was performed with Zen (2012) software (Carl Zeiss, North Ryde, Australia). Some sections were pre-incubated with a 1/20 dilution of α-l-arabinofuranosidase (Megazyme, Ireland) to remove arabinose from the xylan backbone for 60 min and washed before LM11 treatment (Wilson et al., 2012).

### Biochemical assays

Samples for biochemical analysis were frozen in liquid nitrogen and lyophilised (FreeZone, Labconco, MO, USA). Chits (rootlets) were manually removed before grinding (Retsch Mill MM400, Retsch GmbH, Haan, Germany) at 30 Hz for 75 s. Analyses were performed in duplicate or triplicate.

The (1,3;1,4)-β-glucan content of grain was assessed using a small scale version of the Megazyme Mixed-Linkage β-Glucan Assay (McCleary and Codd, 1991) on 15 mg flour samples according to Burton et al. (2011). (1,3;1,4)-β-Glucanase activity was assessed using a small-scale version of the Megazyme Malt and Bacterial Beta-Glucanase & Cellulase assay procedure (Azo-Barley Glucan Method) on 25–50 mg flour samples (McCleary and Shameer, 1987). Starch content was assessed on the alcohol insoluble residue (two washes in 70% ethanol) using a small scale version of the Megazyme Total Starch Assay (amyloglucosidase/α-amylase method) on 40 mg of material (McCleary et al., 1994). Monosaccharide content was assessed on alcohol insoluble residue digested in 1 M H_2_SO_4_ for 3 h at 100°C by reversed-phase high performance liquid chromatography, as described by Burton et al. (2011). Arabinoxylan content was calculated by adding the amount of arabinose and xylose in the hydrolysates and multiplying by 0.88 to allow for the loss of water. Starch, (1,3;1,4)-β-glucan and arabinoxylans contents were calculated as a percentage of the flour weight on a dry basis.

The activity of the alpha-amylase (McCleary et al., 2002) was assayed using a small scale version of Megazyme α-Amylase Assay Kit (Ceralpha Method) on 10 mg flour (McCleary et al., 2002). The activities of both free and total β-amylase were assayed using a small scale version of Megazyme β-Amylase Assay Kit (Betamyl-3) on 25 mg flour (McCleary and Codd, 1989). To measure total limit dextrinase, 25 mg flour was extracted in 400 μL of 0.2 M of sodium acetate (pH 5.0) supplemented with 0.35% l-cysteine, incubated at 40°C for 5 h with interval mixing (Longstaff and Bryce, 1993). The free limit dextrinase was extracted without the use of l-cysteine. Limit dextrinase activity was assayed using the substrate 4,6-*O*-benzylidene-4-nitrophenyl-6^3^-α-d-maltotriosyl-maltotriose (BPNPG3G3) from the Megazyme Pullulanase/Limit-Dextrinase Assay Kit (PullG6 Method) (McCleary et al., 2014).

### RNA extraction and qPCR

Total RNA was extracted from samples comprising two whole grains using the Sigma-Aldrich Spectrum™ Plant Total RNA Kit (Sigma-Aldrich, St Louis, MO) with the addition of a 6-min incubation with thermostable α-amylase (Megazyme, Wicklow, Ireland) in lysis buffer at room temperature prior to addition of β-mercaptoethanol (Betts et al., 2017). Following treatment with TURBO DNase-*free* (Ambion, Life Technologies, Waltham MA), cDNA synthesis was performed using SuperScript®III Reverse Transcriptase according to manufacturer's instructions (Life Technologies, Waltham, MA). Details of gene names, MLOCs and primer details are presented in Table S1. QPCR primers were designed using Primer 3 software (Koressaar and Remm, 2007) and selected based on specificity as determined by blastn software (Table S1; Acland et al., 2013). qPCR was performed as described by Burton et al. (2008) with data normalised using the reference genes *HvCyclophilin, HvGAPdH2, HvHSP70*, and *HvTubulin* (Vandesompele et al., 2002).

## Results

### Grain morphology

**Figure 1:**
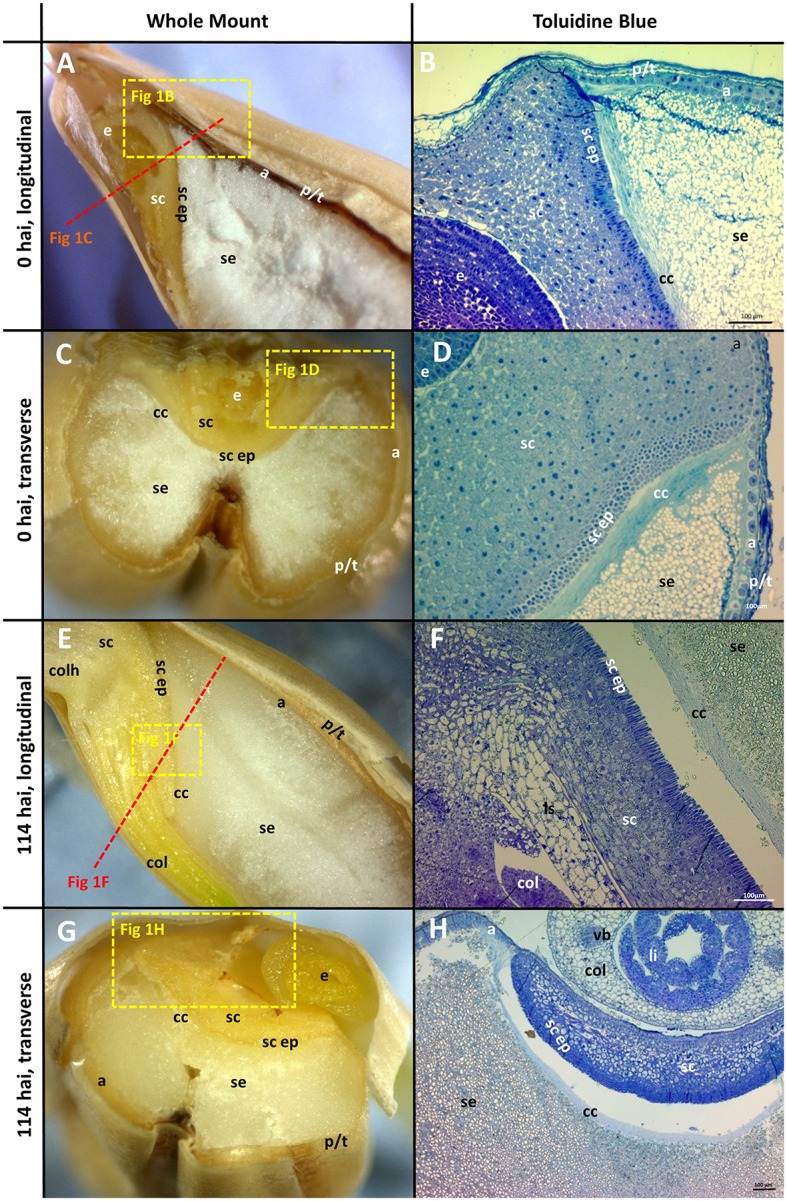
Morphology of the grain at the beginning (0 hai) and end (114 hai) of malting. **(A,C,E,G)** Navigator whole grain sectioned with a razor blade. Dotted lines show indicative positions for other sections. (**B,D,F,H)** Thin sections stained with toluidine blue; **(A,B,E,F)** longitudinal sections; **(C,D,G,H)** transverse sections. **(B,D)** are the variety Admiral and **(F,H)** are Navigator. Scale bars represent 100 μm. a, aleurone; se, starchy endosperm; e, embryo; p/t, pericarp and testa; cc, crushed cell layer; sc, scutellum; sc ep, scutellar epithelium; col, coleoptile; li, leaf initial; vb, vascular bundle; colh, coleorhiza.

**Figure 2:**
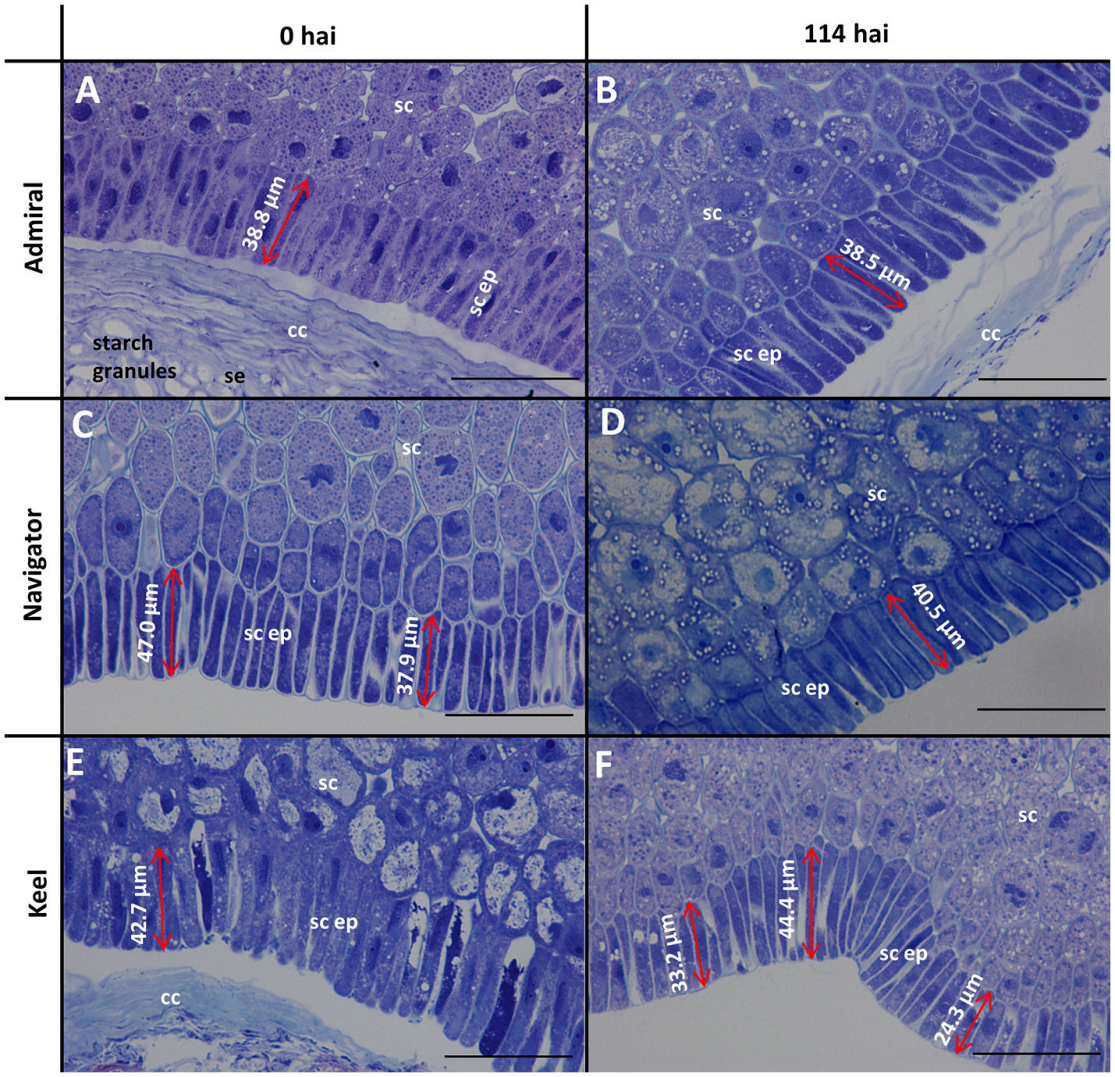
Toluidine blue stained scutellar epithelial cells in mature and germinated grain. **(A,B)** Admiral; **(C,D)** Navigator**; (E,F)** Keel; **(A,C,E)** mature grain (0 hai); **(B,D,F)** 114 hai, Scale bars represent 50 μm. se, starchy endosperm; cc, crushed cell layer; sc, scutellum; sc ep, scutellar epithelium.

The morphology of mature (0 h) and germinated grain (114 h) is shown in Figure 1. The outer layer of maternal tissues, the embryonic tissues and endosperm are clearly distinguishable. As germination progresses the elongation of the embryonic axis becomes obvious (Figures 1E,F). At 114 h after imbibition (hai), the coleoptile can be seen growing down the length of the grain between the aleurone and husk (Figure 1E). Scutellar epithelial cells are observed to be a single layer of elongated, relatively narrow cells that run approximately perpendicular to the scutellum/starchy endosperm interface (Figures 2A,C,E). At 114 hai, the scutellar epithelium cells have separated from each other at the tips, but have not increased substantially in length in any of the varieties examined (Figures 2B,D,F).

The aleurone cells are characterised by relatively thick cell walls and contain a dense matrix of intracellular protein bodies, oil droplets and membrane fractions (Fincher, 1989). In contrast, starchy endosperm cells have much thinner cell walls, and much of their intracellular space is occupied with starch granules, embedded in a protein matrix (Figures 1B,D). Generally, the aleurone layer in barley is two to three cells thick but can vary from one to four cells depending on the location in the grain (Figure 1B). The layer becomes thinner at the embryo end of the grain, such that the aleurone is only a single cell thick once it reaches the scutellum (Figure 1D). The aleurone layer was also observed to continue proximal to the endosperm between the scutellum and the pericarp/testa (Figure 1D; Brown and Morris, 1890; Bacic and Stone, 1981b).

**Figure 3:**
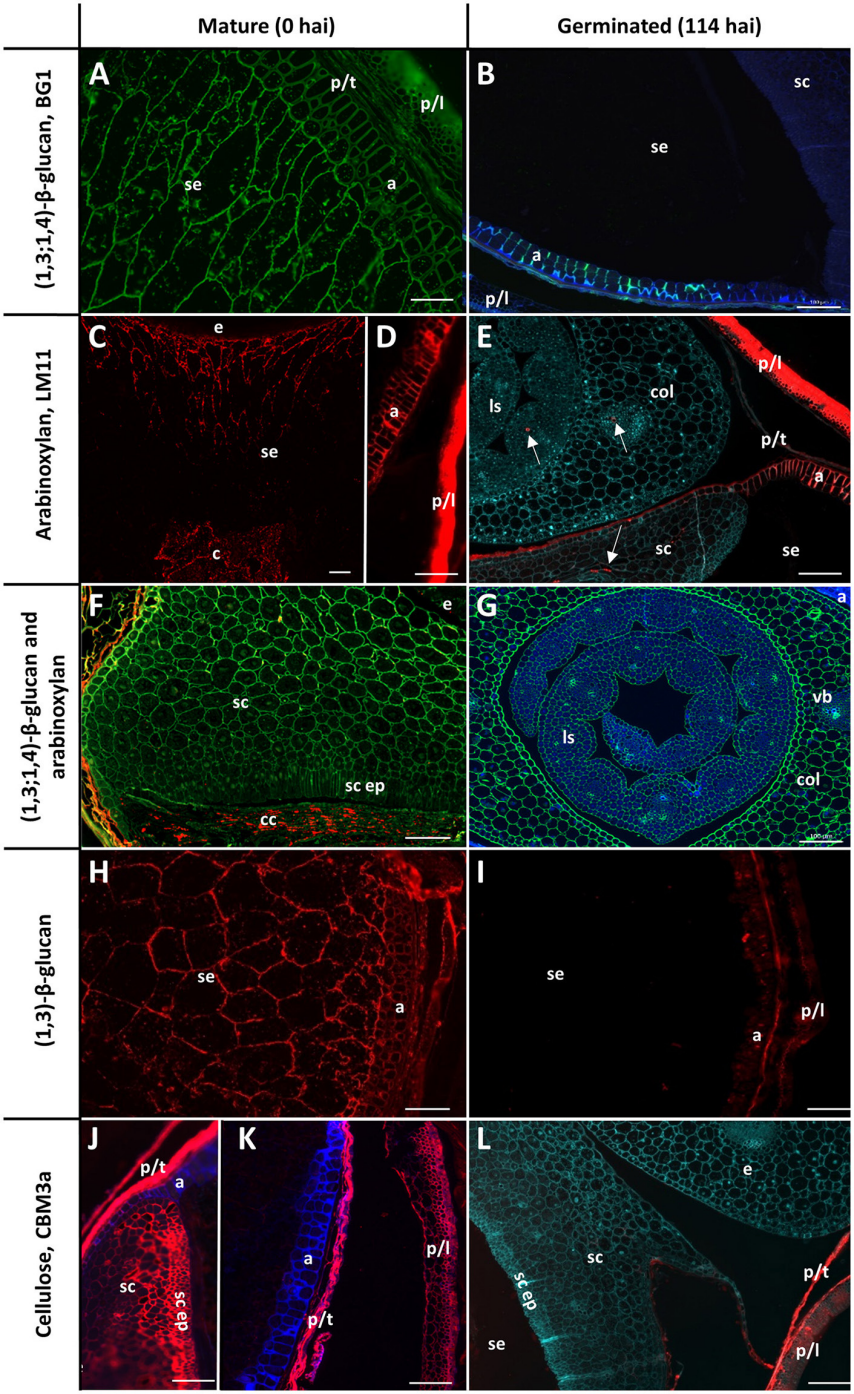
Fluorescent immuno-histochemical analysis of cell wall polysaccharides in transverse sections of ungerminated (0 hai, left) and germinated (114 hai, right) grain. **(A,B,F,G)** Detection of β-glucan by the antibody BG1 (green), blue shows auto-fluorescence. **(C–F)** Detection of arabinoxylan with the antibody LM11 (red). **(E)** was pretreated with α-l-arabinofuranosidase prior to LM11 binding, turquoise shows Calcofluor counter staining, arrows indicate pockets of arabinoxylan label. **(F)** shows double labelling of the embryo with both BG1 (green) and LM11 (red). **(H,I)** Detection of callose with the (1,3)-β-glucan antibody (red). **(J–L)** Cellulose is labelled in red using CBM3a; blue shows auto-fluorescence; turquoise shows Calcofluor counter staining. **(A,C–E,G)** Navigator; **(B,F,H,I–L)** Admiral. Scale bars represent 100 μm. Negative controls are shown in Figure S1. a, aleurone; col, coleoptile; c, crease; cc, crushed cell layer; e, embryo; ls, leaf sheath; p/l, palea and lemma; p/t, pericarp and testa; se, starchy endosperm; sc ep, scutellar epithelium; sc, scutellum.

The cell walls in the starchy endosperm of ungerminated barley consist mainly of (1,3;1,4)-β-glucan (Figure 3A) with only small amounts of arabinoxylan detected by immuno-histochemistry (Figure 3C). Direct comparisons of staining intensities of cell wall polysaccharides with different antibodies, however, is generally not possible because of the different affinities of the antibodies for their antigens. Endosperm cell walls are thin compared with other cell walls in the grain, particularly the thick aleurone cell walls that consist predominantly of arabinoxylan (Figure 3D). The cell walls of the scutellum and embryo also contain (1,3;1,4)-β-glucans but no detectable arabinoxylan (Figure 3F). An antibody against (1,3)-β-glucan (callose) revealed the presence of deposits of callose in the cell wall regions of the starchy endosperm (Figure 3H). Strong labelling of cellulose by the carbohydrate binding module CBM3a was found in the pericarp, testa, palea, lemma, scutellum, and scutellar epithelium (Figures 3J,K).

By the end of the simulated malting process, strong labelling of (1,3;1,4)-β-glucans by BG1 remained in the aleurone and embryo, but labelling in the starchy endosperm had almost completely disappeared (Figures 3B,G). By 114 hai, strong labelling of arabinoxylans remained in the maternal tissues and aleurone layer but no labelling was detected in the starchy endosperm (Figure 3E). Additionally, small pockets of arabinoxylan labelling were observed in the coleoptile and leaf tissue associated with developing vascular bundles when the sections were pre-incubated with α-l-arabinofuranosidase (Figure 3E): this unmasking was required to generate the correct epitope for the LM11 antibody, which binds arabinoxylans with low levels of substitution (McCartney et al., 2005), and does not indicate endogenous AXAH activity. These tissues were heavily labelled by BG1 (Figure 3G). Only small pockets of (1,3)-β-glucans remained in the aleurone layer at 114 hai and no labelling was observed in the starchy endosperm (Figure 3I). Labelling of cellulose by CBM3a remained in the pericarp, testa, palea, lemma but not in the scutellum or scutellar epithelium (Figure 3L).

**Figure 4:**
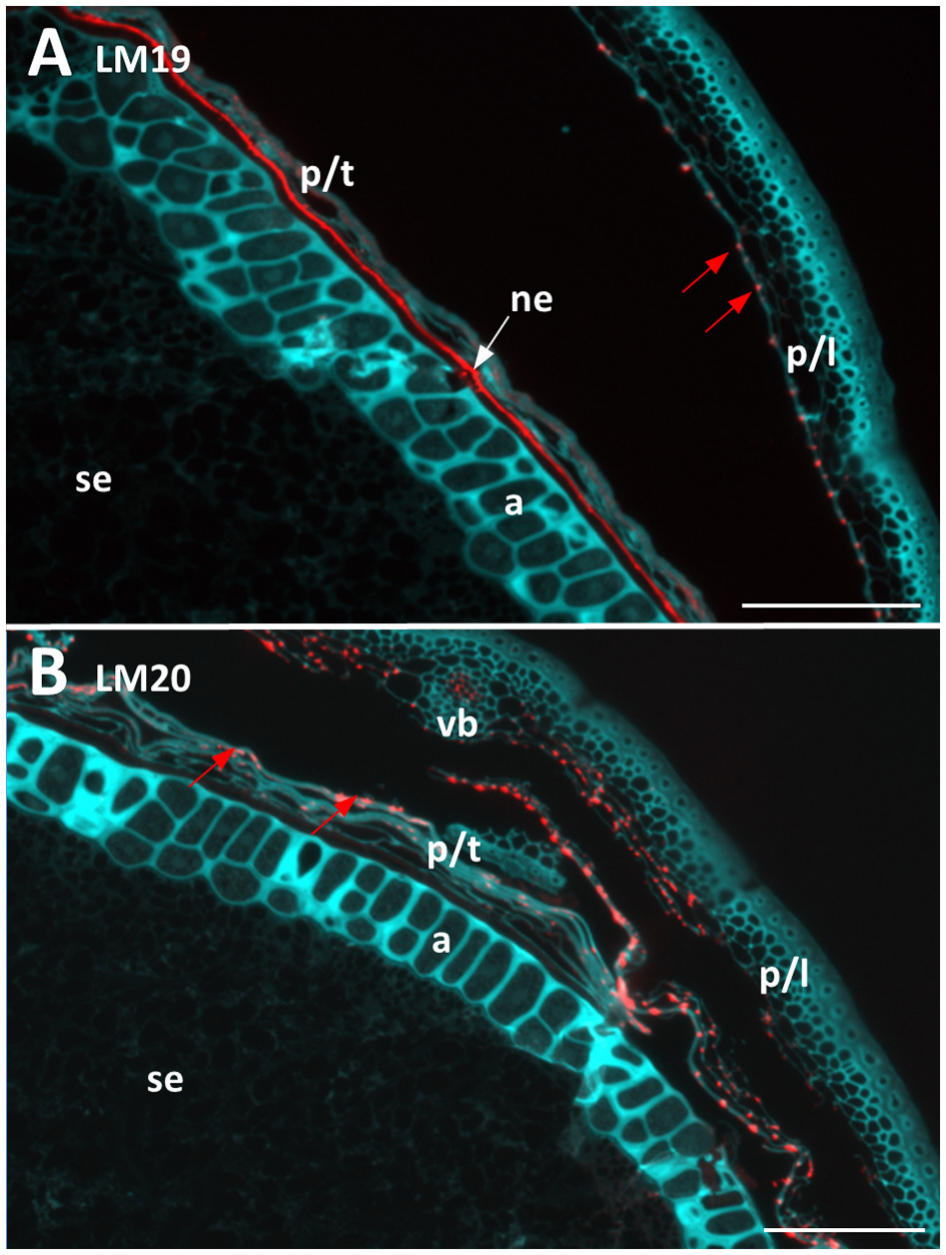
Fluorescent immuno-histochemical analysis of pectic cell wall polysaccharides in transverse sections of ungerminated (0 hai) Navigator grain. **(A)** Un-esterified homogalacturonan pectin labelled with LM19 (red), turquoise shows auto-fluorescence. **(B)** Methyl esterified homogalacturonan pectins labelled with LM20 (red), turquoise shows auto-fluorescence. Red arrows indicate pockets of label. Scale bars represent 100 μm. a, aleurone; ne, nucellar epithelium; p/l, palea and lemma; p/t, pericarp and testa, se, starchy endosperm; vb, vascular bundle.

Two antibodies were used to locate pectic polysaccharides (Verhertbruggen et al., 2009). Labelling of un-esterified homogalacturonan by LM19 was found in a single layer in the nucellar epithelium and in small deposits in the palea and lemma (Figure 4A). LM20 labelled methyl-esterified homogalacturonans in the palea and lemma in a punctate fashion, with a small amount of labelling observed in the pericarp (Figure 4B). No labelling was observed in the starchy endosperm or aleurone layer by either antibody.

### Starch hydrolysis

**Figure 5:**
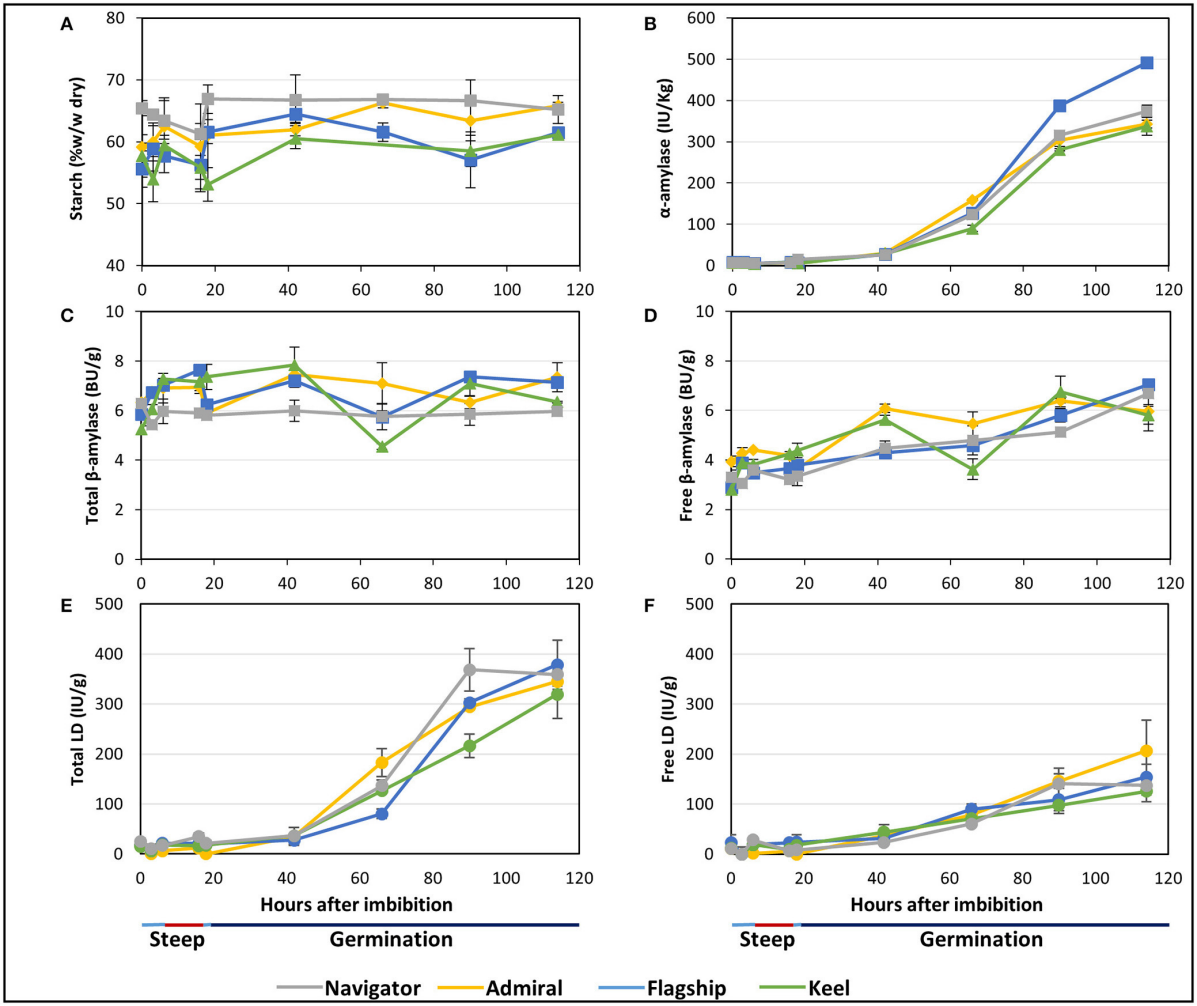
The starch content and starch degrading enzyme activities in barley grain during a simulated malting process. **(A)** Starch content of alcohol insoluble grain material. **(B)** α-amylase activity. **(C)** Activity of total β-amylase in the grain. **(D)** Activity of free β-amylase in the grain. **(E)** Activity of total limit dextrinase (LD) in the grain. **(F)** Activity of free LD in the grain.

Starch is the major carbohydrate present in mature barley grain, contained predominantly within the endosperm. As expected, the starch content as a proportion of flour weight remained constant or increased throughout malting. Not only are these elite lines selected to maximise starch levels in malt and minimise starch malting losses, but significant amounts of grain material, such as the rootlets and soluble sugars and proteins, were removed before analysis causing the overall starch content, measured as a percentage of flour weight, to remain constant or to increase during malting. Navigator had the highest starch content at maturity and throughout the malting process (65.4–65.2% w/w) (Figure 5A). Initial amounts in Admiral, Flagship and Keel were lower (59.1, 55.6, and 57.7% w/w, respectively), which increased to 65.8, 61.5, and 61.1% of the final weight, respectively, by 114 hai (Figure 5A).

Total α-amylase activity followed similar patterns in the four barley cultivars, starting at negligible levels during steeping, and increasing throughout germination to peak levels at 114 hai (Figure 5B). Enzyme activity was highest in Flagship, with approximately 30% higher α-amylase activity than other varieties by 114 hai.

The amount of total β-amylase enzyme activity did not increase during the simulated malting process in any of the varieties, however free β-amylase activity increased from the end of the second steep (18 hai; Figures 5C,D).

Total limit dextrinase (LD) activity remained steady at low but detectable levels throughout steeping, but increased sharply from 42 hai and peaked at 114 hai (Figure 5E). Free LD activity followed a similar trend, contributing to approximately 50% of the total activity by 90 hai (Figure 5F).

### Cell wall hydrolysis

#### (1,3;1,4)-β-glucan and other glucans

**Figure 6:**
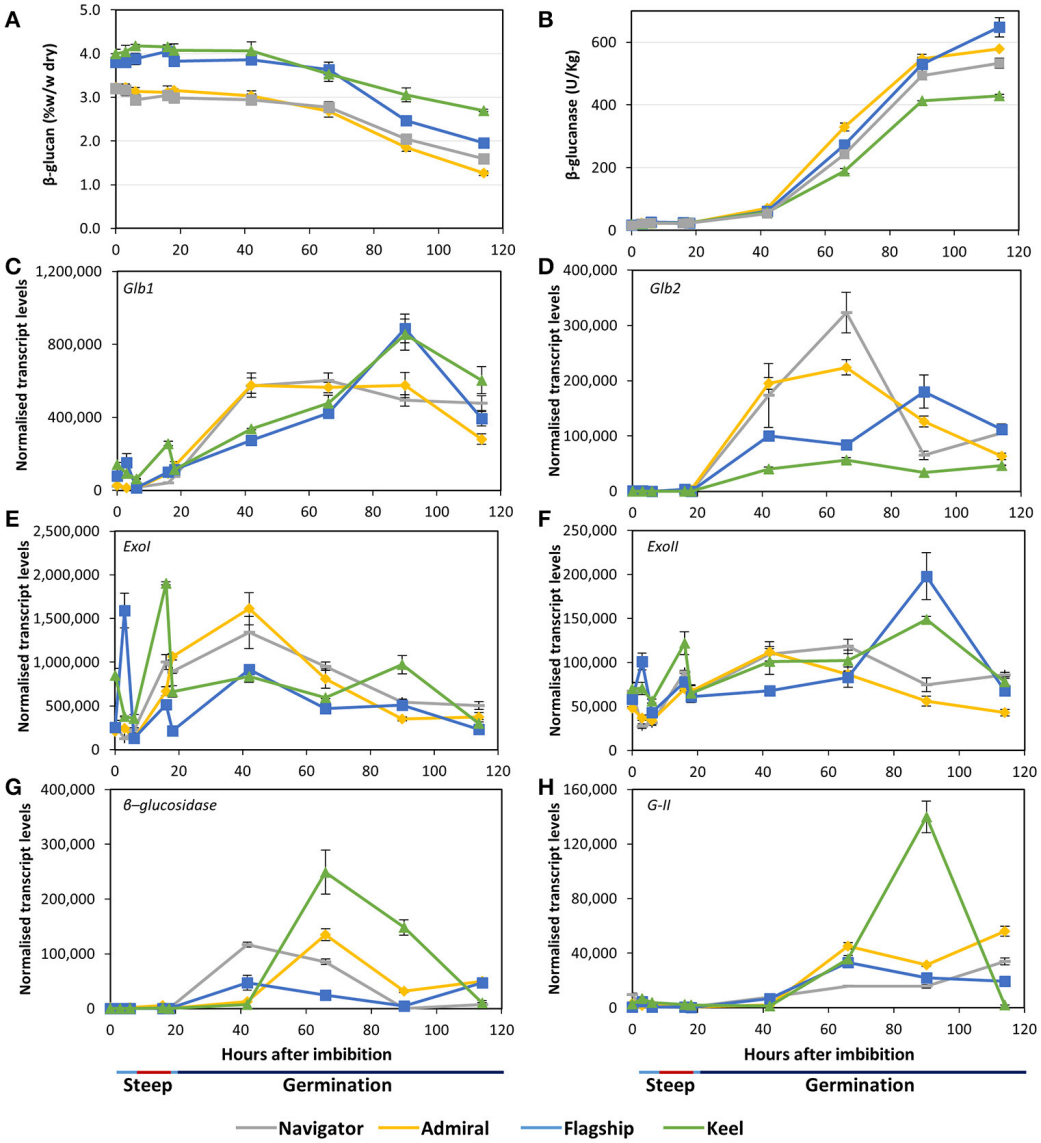
**(A)** (1,3;1,4)-β-glucan content and **(B)** (1,3;1,4)-β-glucanase enzyme activity in barley grain during a simulated malting process. Gene expression levels of **(C,D)** (1,3;1,4)-β-glucanase genes *Glb1* and *Glb2*; (**E,F)** (1,3)-β-glucan exohydrolase *ExoI* and *ExoII*; **(G)** β-glucosidase; and **(H)** β-glucan glucohydrolase *G-II*. QPCR units are normalised transcript levels (arbitrary units), error indicates standard deviation of three experiments.

Initial (1,3;1,4)-β-glucan content ranged from 3.2% in Admiral and Navigator to 4.0% in Keel (Figure 6A). During the simulated malting, the levels declined from 42 hai onwards in all cultivars, dropping to final levels of 1.3–2.7%. Correspondingly, all cultivars exhibited very low total (1,3;1,4)-β-glucanase activity during steeping (Figure 6B), with activity increasing from the first 24 h of germination to reach final levels of 10 times the initial levels by the end point (114 hai). The feed variety Keel had both the smallest reduction in (1,3;1,4)-β-glucan content and the lowest (1,3;1,4)-β-glucanase activity.

Transcript levels of the two (1,3;1,4)-β-endoglucanase *Glb1* and *Glb2* genes (isoenzymes EI and EII) remained relatively low throughout steeping but increased quickly from 18 hai, the onset of the germination phase (Figures 6C,D). *Glb1* transcript levels were consistently higher than *Glb2* levels. Transcript levels of both genes reached their maxima earlier in Admiral and Navigator than in Flagship or Keel, however levels of *Glb1* were higher in Flagship and Keel by 90 hai.

Also involved in (1,3;1,4)-β-glucan hydrolysis are β-glucan glucohydrolases and β-glucosidase. Two members of the β-glucan glucohydrolase family, namely genes encoding isoenzymes *ExoI* and *ExoII* (Hrmova et al., 1996; Harvey et al., 2001), were investigated, along with a gene encoding β-glucosidase (MLOC_37740). Both β-glucan glucohydrolase genes were transcribed at high levels throughout the simulated malting process (Figures 6E,F), with *ExoI* transcripts being approximately 10 times higher than those for *ExoII*. Expression in Keel and Flagship peaked during steeping for both genes and were still high later in germination. In contrast, the transcript levels in varieties Admiral and Navigator started at relatively low levels and peaked at 42 hai (24 h into germination). Transcript levels for β-glucosidase were highest for Keel, peaking at 66 hai, and lowest for Flagship (Figure 6G). The increases in transcript levels of *Glb1, Glb2*, and β-glucosidase coincided with the increase in (1,3;1,4)-β-glucanase activity and the decrease in (1,3;1,4)-β-glucan content.

After finding callose in mature grain (Figure 3H), transcript levels of β-glucan glucohydrolase gene isoenzyme *G-II* were also investigated (Xu et al., 1992). Transcript levels increased significantly after 42 hai in all varieties (Figure 6H). However, the feed variety Keel produced four times the level of transcript by 90 hai compared with the three malting varieties.

#### Arabinoxylan

**Figure 7:**
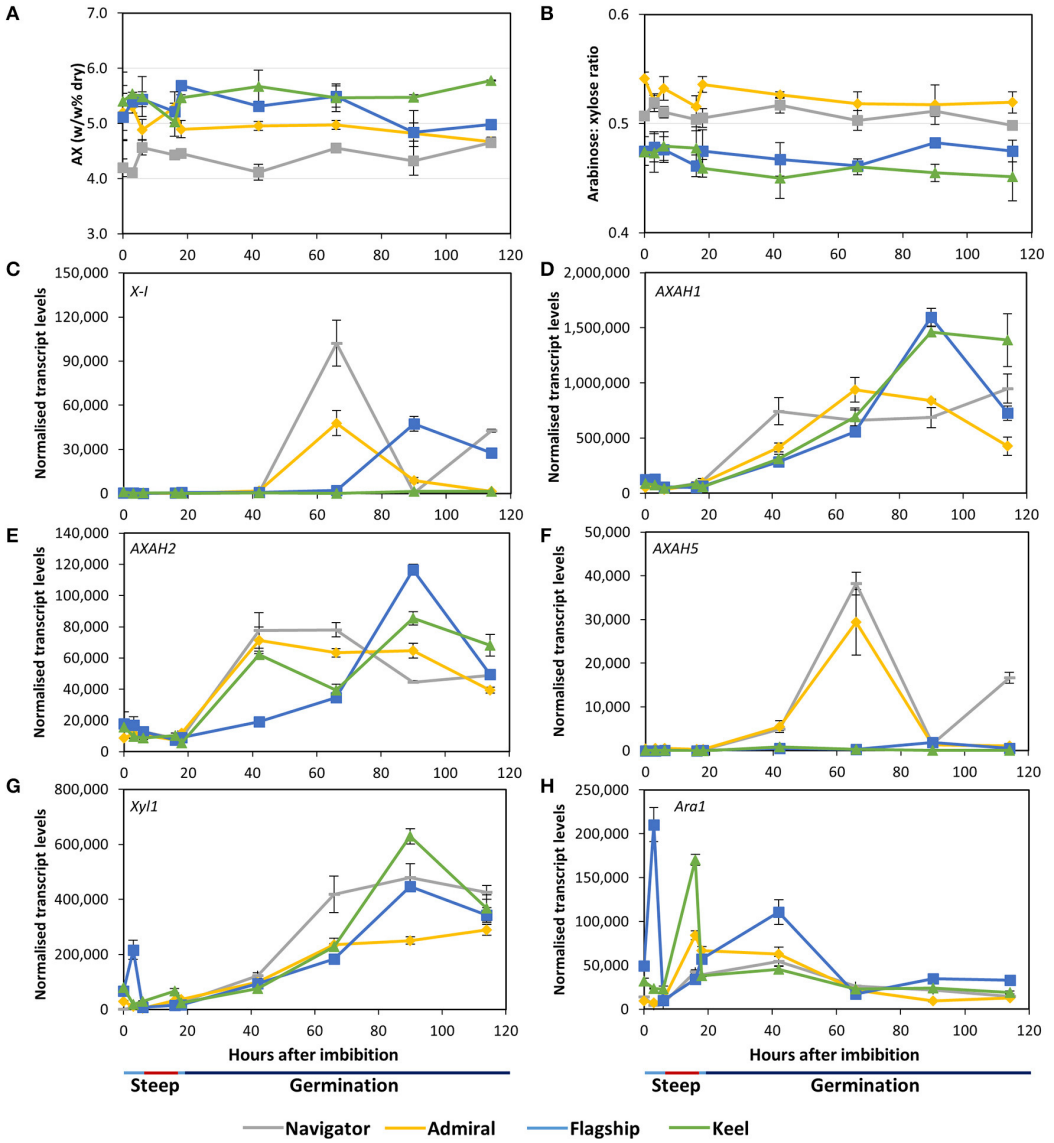
**(A)** Total arabinoxylan content of alcohol insoluble grain material and **(B)** arabinose: xylose ratio in barley grain during a simulated malting process. Gene expression levels of **(C)** (1,4)-endoxylanase I; (**D–F)** arabinose arabinofuranosidase 1, 2, and 5; (**G)** xylosidase; and **(H)** arabinofuranosidase. QPCR units are normalised transcript levels (arbitrary units), error indicates standard deviation of three experiments.

Arabinoxylan content remained relatively stable throughout the malting time-course in all cultivars (Figure 7A). Keel maintained the highest arabinoxylan content, followed by Flagship, Admiral, and Navigator. The arabinose to xylose ratio of 0.45–0.55 did not vary over time (Figure 7B).

Of the endoxylanase genes examined, *X-I* transcript levels were highest (Figure 7C), remaining low during steeping but rising sharply from 42 hai. Navigator had the highest transcript levels, double those detected in Admiral and Flagship, while levels in Keel remained low throughout the time-course. Transcript levels of *X-II* and *X-III* genes were low throughout the simulated malting process, although *X-II* exhibited a peak in transcription at 42 hai in all four varieties (Table S2).

Transcript levels of *AXAH1* were relatively low during steeping and increased during the early stages of the germination phase (Figure 7D), with peak amounts varying by cultivar after 66 hai. Admiral and Navigator *AXAH1* transcripts peaked earlier than, but at a level approximately half that of, Flagship and Keel. Transcript levels of *AXAH2* followed a similar trend to *AXAH1* but at much lower levels (Figure 7E). Levels of *AXAH3* and *AXAH4* remained low throughout the simulated malting (Table S2). Transcript levels of *AXAH5* were lower than for *AXAH2*, and showed a single peak at 66 hai in Admiral and Navigator but not in Keel or Flagship (Figure 7F).

*Xyl1* transcripts remained low throughout steeping and increased during the first 24 h of the germination phase (Figure 7G). Transcript levels of *Ara1* were relatively low, fluctuating during the simulated malting process, peaking at 3 hai for Flagship, 16 hai for Keel and Admiral, and 42 hai for Navigator, and generally declining toward the end of the time course (Figure 7H).

## Discussion

### Cell wall changes

The largest difference between varieties was in their levels of grain (1,3;1,4)-β-glucan, which dropped during the simulated malting process but much less in Keel than in the malting varieties. High levels of residual (1,3;1,4)-β-glucan in malt can lead to filtration difficulties during brewing, and haze formation in the final product (Bamforth, 1985). The low (1,3;1,4)-β-glucan contents of Flagship, Navigator, and particularly Admiral, by the end of the malting time-course (Figure 6A) positively reflect the efforts of breeding programs to reduce levels of (1,3;1,4)-β-glucan in mature barley grain, or to maximise levels of (1,3;1,4)-β-glucanases during malting. (1,3;1,4)-β-Glucan in mature barley grain is located predominantly in the cell walls of the starchy endosperm, scutellum, and embryo (Figures 3A,F, Fincher, 1975; Bacic and Stone, 1981b). By 114 hai, these scutellum and endosperm cell walls had degraded almost completely and contained no polysaccharides detectable by immuno-histochemical analysis, suggesting that endosperm cell wall modification was complete at the end of the simulated malt.

The two (1,3;1,4)-β-endoglucanases EI and EII, encoded by genes *Glb1* and *Glb2*, respectively, are primarily responsible for (1,3;1,4)-β-glucan hydrolysis in germinating grain (Slakeski and Fincher, 1992). Substantial increases in *Glb1* and *Glb2* transcript levels within the first 24 h after steeping correlated with increased total (1,3;1,4)-β-glucanase activity and a decline in (1,3;1,4)-β-glucan content (Figures 6A–D), as confirmed by fluorescent immuno-histochemical microscopy (Figure 3B). Earlier and higher levels of *Glb1* transcript (Figures 6C,D) may be due to the restriction of *Glb2* transcription to the aleurone, while *Glb1* is also expressed in the scutellum (Slakeski and Fincher, 1992). The high β-glucanase levels at 114 hai were found in Flagship and are likely due to a combination of EI and EII enzyme activity: higher levels of EI than Navigator and Admiral, and higher levels of EII than Keel. While past research has indicated that EII may be more important for brewing due to its higher thermostability and faster hydrolysis rate (Woodward and Fincher, 1982), the three-fold higher transcript levels of *GlbI* suggest an important role for EI in malting. Although transcript levels do not necessarily correlate directly with enzyme activity levels, *GlbI* may be a profitable target for improvement in future breeding programs.

We have also confirmed that significant, persistent callosic deposits are present in the starchy endosperm of mature grain (Figure 3H). Callose has previously been detected in developing endosperm cell walls in barley, wheat, and rice (Fulcher et al., 1977; Wood and Fulcher, 1984; Brown et al., 1996; Wilson et al., 2006, 2012; Palmer et al., 2015), mainly associated with plasmodesmata late in grain development (Wilson et al., 2012), while Palmer et al. (2015) suggested that callose may be important in the differentiation of aleurone cells into sub-aleurone cells in wheat. It has also been suggested that the callose present in the endosperm is due to a wound response as the grain fills and matures, which might cause the plasma membrane to become detached from the cell wall with the concomitant deposition of callose (Wilson et al., 2012). The highly variable amounts of callose found in cereal grains may be due to callose deposition as a result of transient moisture stress during grain development (Fincher, 1989). The callose is likely degraded by β-glucan glucohydrolase enzymes; transcript levels of β-glucan glucohydrolase isoenzyme *G-II* increased during germination in all cultivars, especially Keel, although other β-glucan glucohydrolases may also play a role in germination given the large size of the gene family (Xu et al., 1992; Li et al., 1996). As the callose had been fully hydrolysed during malting in these varieties (Figure 3I), it may be worth considering callose as a potential contributor to glucose content in malt and wort. Transcript levels of β-glucan glucohydrolases *ExoI* and *ExoII*, which degrade oligosaccharides from hydrolysed (1,3;1,4)-β-glucan and (1,3)-β-glucan (Hrmova et al., 2002; Fincher, 2009), were very high in all varieties throughout the malting process (Figures 6E,F). The reason for their high transcript levels very early in germination—before expression of most of the endohydrolases—is unknown, and suggests a role for these exo-acting enzymes in the later stages of grain development. It has been suggested that the (1,3)-β-glucan endohydrolases might provide protection to the germinated grain against pathogen invasion, given that these enzymes can hydrolase the (1,3)-β-glucan and (1,3;1,6)-β-glucans of fungal cell walls (Fincher, 1989).

Arabinoxylan in mature barley grain is present predominantly in the aleurone cell walls and maternal tissues, with lower levels in the starchy endosperm (Figures 3C,D). Small pockets of arabinoxylan labelling were found in the embryo at 114 hai (Figure 3E; Wilson et al., 2012), primarily located in developing vascular tissue of the developing leaf sheath and coleoptile. Throughout the simulated malting process, there were no significant changes detected in arabinoxylan content or structure either biochemically or microscopically (Figures 3E, 7A,B), probably due to preponderance of arabinoxylans from maternal tissues, which remain unchanged during germination. However, changes were observed in transcript levels of genes involved in arabinoxylan modification and hydrolysis (Figures 7C–H); increases in transcript levels are likely due solely to changes within the living aleurone and embryonic cells. Only *AXAH2* has previously been detected in developing coleoptiles (Laidlaw et al., 2012), suggesting that *AXAH1* and *5* may be expressed in other tissues such as the aleurone. Given the clear presence of *AXAH1, 2*, and *5* transcripts and reports of AXAH activity in grain tissues (Sungurtas et al., 2004), examination of isolated aleurone cells may reveal information about changes in arabinoxylan content and/or structure that cannot be detected in whole grain extracts.

Modification of arabinoxylan structure by xylanase or xylosidase enzymes was not detected, either microscopically or biochemically, suggesting that within the time constraints of this experiment, these enzymes were not sufficiently active to produce short, soluble oligosaccharides that would be removed during preparation of alcohol insoluble residue. The temporal and spatial details of endoxylanase synthesis and secretion are not well-understood; it has been suggested that endoxylanase enzymes, active or bound, are not released from the aleurone cells until after cell death (Fincher, 1989; Slade et al., 1989; Caspers et al., 2001; Simpson et al., 2003; Van Campenhout and Volckaert, 2005). Our observations are consistent with the late release of active endoxylanase and xylosidase enzymes from the aleurone into the starchy endosperm, after the 114 h of this time course.

None of the outer, maternal tissues appeared to undergo compositional changes through the simulated malting process. Immuno-histochemical analysis confirmed the presence of cellulose, arabinoxylan, and (1,3;1,4)-β-glucans in these tissues (Figure 3; MacLeod and Napier, 1959), and revealed small amounts of mainly methyl esterified homogalacturonan pectin, in a punctate distribution (Figure 4B), and un-esterified homogalacturonan pectin in the nucellar epithelium (Figure 4A). Similar observations were made in developing wheat and rice grains (Chateigner-Boutin et al., 2014; Palmer et al., 2015). Recently, the presence of methyl-esterified homogalacturonan was also detected in wheat endosperm after the enzymatic removal of (1,3;1,4)-β-glucan and arabinoxylan (Chateigner-Boutin et al., 2014); while not detected in this work (Figure 4), it is possible that pectin is present in the endosperm and aleurone but masked by other cell wall components (Fincher, 1975; Bacic and Stone, 1981a; Xue et al., 2013).

### Starch hydrolysis

Enzymes involved in starch depolymerisation were detected in abundance in the barley grain during simulated malting. Total α-amylase activity followed similar patterns in the four barley cultivars, starting at negligible levels during steeping, and increasing throughout germination (Figure 5B). Enzyme activity was approximately 30% higher in Flagship, compared with the other varieties. At this stage, we have not undertaken a comprehensive analysis of transcripts of starch hydrolysis genes as the recently revised barley genome contains a much larger number of α-amylase genes than had been previously identified (Mascher et al., 2017). The identification of the spatial and temporal expression patterns of specific genes involved in the starch degradation process during germination remains an important and complex research target.

Following grain imbibition, bound β-amylase is released by proteolytic activity so that both active and inactive β-amylase are present in germinating grain. Total β-amylase activity was found to remain constant during the simulated malting process while the amount of free β-amylase activity increased from approximately 50–95% of the total by the end of malting (Figures 5C,D). These findings suggest that the increase in β-amylase activity observed after germination is solely due to activation of β-amylase already present in the grain rather than due to additional *de novo* synthesis during germination.

Like β-amylase, LD is produced during grain development and held inactive but the gene is also transcribed during germination. However, by the end of malting, free LD activity only represented about half of the total LD activity in the grain (Figures 5E,F), and previous reports suggest that approximately 70% of the enzyme present in the grain is bound to the limit dextrinase inhibitor throughout malting (Longstaff and Bryce, 1993; Sissons et al., 1993; Burton et al., 1999; Ross et al., 2003). Given the importance of amylopectin hydrolysis during germination (Naka et al., 1985), understanding the spatial expression and interactions of limit dextrinase and limit dextrinase inhibitor remains central to our ability to improve the efficiency of starch hydrolysis in malting cultivars.

### Morphology of the aleurone and scutellum

Scutellar cells are generally spherical, but scutellar epithelial cells are elongated perpendicular to the crushed cell layer. These cells are about 30–50 μm in length at grain maturity (Figures 2A,C,E; Gram, 1982), and separate to increase the surface area closest to the endosperm by 114 hai (Figures 2B,D,F). No evidence was found that these cells had elongated significantly by the end of the simulated malting process (Figure 2), in contrast to previous studies showing that scutellar epithelium cells in germinated wheat and barley grains elongated to twice their original length by 72 h after imbibition (Brown and Morris, 1890; MacLeod and Palmer, 1966; Gram, 1982). Additionally a large variation in length of the scutellar epithelial cells was observed in the feed variety Keel at 114 hai (Figure 2F), which may be due to the commencement of the elongation process. Whether this difference is due to the controlled environment of the malting process or varietal differences is unknown. It would be interesting to examine whether the morphology of this critical secretory and absorptive organ has changed due to selection for elite malting qualities.

While both scutellum and aleurone cells play a secretory role during germination, their fates as germination progresses are quite different (Fincher, 2010). The differences in cell wall composition in these tissues, and the way they change during germination, may reflect their dissimilar final roles in germination, rather than their common role at the beginning. Arabinoxylan and (1,3;1,4)-β-glucan, the major cell wall polysaccharides in aleurone cells, are still detected at the end of the simulated malting, long before cell death occurs (Figures 3B,E; Bacic and Stone, 1981a). In contrast, (1,3;1,4)-β-glucans and cellulose of the scutellum cell walls, are almost completely degraded by the end of malting (Figures 3B,L). McFadden et al. (1988) showed that (1,3;1,4)-β-glucanase genes were transcribed initially in the scutellar epithelium and that transcription moved along the aleurone layer from the proximal to the distal end of the grain, so (1,3;1,4)-β-glucan in the scutellum would be hydrolysed before that in the aleurone.

## Conclusion

We have analysed a comprehensive suite of genes and enzymes known to be important for malting in four barley cultivars and described some novel findings regarding the morphology and composition of cell walls during germination. Overall, Navigator and Admiral exhibited very similar expression patterns for most genes and enzymes, including (1,3;1,4)-β-endoglucanases, β-glucan glucohydrolases, endoxylanases, and AXAHs, while Flagship and Keel also had related expression patterns. However, Flagship differed from Keel in a few crucial genes and enzymes, such as (1,3;1,4)-β-endoglucanase isoenzyme EI and endoxylanase isoenzyme X-1, which may partially contribute to its vastly superior malting qualities. These important enzymes along with other cell wall degrading enzymes, such as (1,3)-β-glucanases may potentially be breeding targets for improved malting quality. Additionally, examination of the new barley genome is revealing many new members of gene families involved in starch hydrolysis (Mascher et al., 2017), suggesting that the numbers of, and interactions between, the enzymes encoded by these genes is likely to be more complicated than previously thought. Improvements in analytical techniques will continue to provide new information about the morphology, composition, and function of different tissues within the grain during malting and germination.

## Author contributions

NB and HC designed and supervised the study and wrote the manuscript; HC and LW performed the microscopy; LW and SK performed the malting and biochemical analyses; LW and NB determined qPCR targets and designed primers; NS performed the qPCR; BS, RB, and GF obtained the research funding; FL, BS, RB, and GF and gave critical suggestions on manuscript preparation. All authors have read and approved the manuscript.

### Conflict of interest statement

FL and BS were employed by the Carlsberg Research Laboratory. Other authors declare that the research was conducted in the absence of any commercial or financial relationships that could be construed as a potential conflict of interest.

## Abbreviations

AXAH: arabinoxylan arabinofuranohydrolase
hai: hours after imbibition
LD: limit dextrinase
qPCR: quantitative real-time polymerase chain reaction.
