# Supplementary figures and images for: Morphology, Carbohydrate Distribution, Gene Expression, and Enzymatic Activities Related to Cell Wall Hydrolysis in Four Barley Varieties during Simulated Malting
Source: Front Plant Sci. 2017 Oct 30;8:1872. doi: 10.3389/fpls.2017.01872 (PMC5670874; doi:10.3389/fpls.2017.01872)

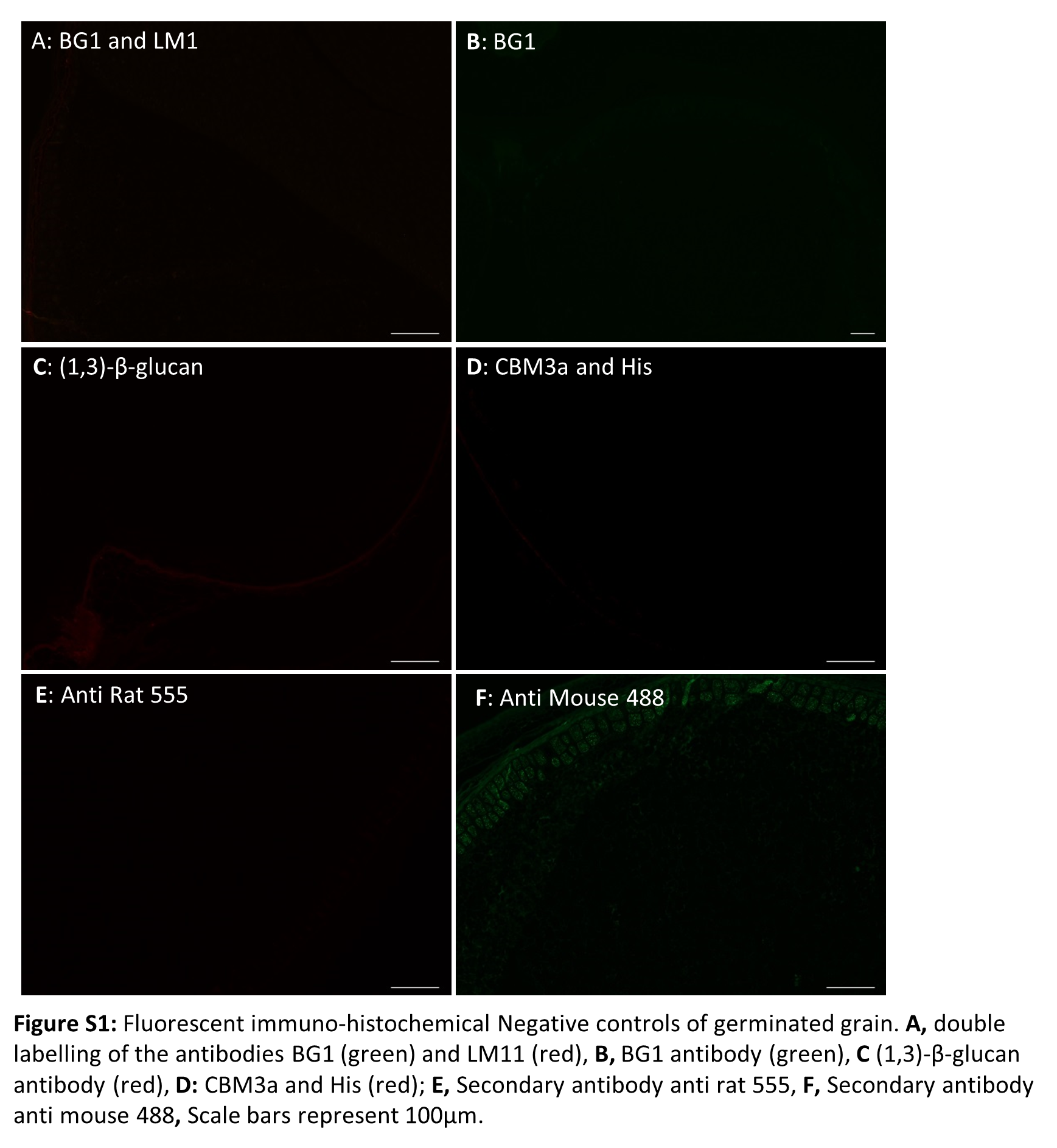

Supplement: Supplementary file 1 [file Image1.tif]
