# Supplementary material for: Morphology, Carbohydrate Distribution, Gene Expression, and Enzymatic Activities Related to Cell Wall Hydrolysis in Four Barley Varieties during Simulated Malting
Source: Front Plant Sci. 2017 Oct 30;8:1872. doi: 10.3389/fpls.2017.01872 (PMC5670874; doi:10.3389/fpls.2017.01872)
Supplement: Supplementary file 2 [file Table1.docx]

**Table S1** Genes and primer details of qPCR analysis.

| Gene Name | Protein Name | MLOC | Size (bp) | CAZy ID | Forward Primer (5' to 3') | Reverse Primer (5' to 3') | Reference |
| --- | --- | --- | --- | --- | --- | --- | --- |
| *Glb1* | (1,3;1,4)-β-endoglucanase EI | MLOC_62746 | 122 | GH17 | AACGAGAACCAGAAGGACAAC | TACGGACATACGGGCACTA | Slakeski et al. (1990) |
| *Glb2* | (1,3;1,4)-β-endoglucanase EII | MLOC_68815 | 253 | GH17 | CTACAACCAGCACCTCATCAA | CGAGTAGCTCGTCAAGTTCG | Slakeski et al. (1990) |
| *G-II* | β-glucan glucohydrolase | MLOC_73077 | 219 | GH17 | TCGCCATGTTCAACGAGAACC | ATGCTTGGTTGCACTCTTCCAT | Xu et al. (1992) |
| *ExoI* | β-glucan exohydrolase I | MLOC_39594 | 130 | GH3 | GGAGATAGGAAAACTGGGGACA | CAGCAACAGTAAGAAAGAATAGCAG | Harvey et al. (2001) |
| *ExoII* | β-glucan exohydrolase II | MLOC_165 | 142 | GH3 | TCGGGTGAGATGGGATGT | CAACAGAACCAACACGACAAC | Hrmova et al. (1996) |
| *β-glucosidase* | β-glucosidase | MLOC_37740 | 330 | GH1 | ACGGGCCATTACCCACAG | CAGGTTGTTTGATTCAGAGGT | Shirley, unpublished |
| *X-I* | xylanohydrolase I | MLOC_73983 | 201 | GH10 | ACTTCAAGTTCAGGGGCTTCT | TTATCCCTTGACGTGTTGCAT | Banik et al. (1996) |
| *X-II* | xylanohydrolase II | MLOC_24879 | 207 | GH10 | ACACTCCTCTCCTGGTGGATT | CGACCCACACAGATTTAGCAT | Banik et al. (1996) |
| *X-III* | xylanohydrolase III | none | 204 | GH10 | AAAGGGGACAACACACCTCTC | TTCACAACACTTGGGCAAAAT | Banik et al. (1997) |
| *AXAH1* | arabinoxylan arabinofuranosidase 1 | MLOC_56099 | 206 | GH51 | GCGGTGTAATGGAGCAGAAC | CTGCCCATCGTTCCCTTATT | Lee et al. (2001) |
| *AXAH2* | arabinoxylan arabinofuranosidase 2 | MLOC_44256 | 248 | GH51 | GCTGGGGTCAAACGCTACT | CGCCTCGTGTATTAATCCTGTT | Laidlaw et al. (2012) |
| *AXAH3* | arabinoxylan arabinofuranosidase 3 | MLOC_10876 | 189 | GH51 | ATGAACCAGGGACGAGAACTT | TCCTCACTCTTTCACTTCATT | Laidlaw et al. (2012) |
| *AXAH4* | arabinoxylan arabinofuranosidase 4 | MLOC_64388 | 177 | GH51 | TGTGAATACAGAAAGGAATGCGATAG | TGAGAGTTAGACCAAGGC | Laidlaw et al. (2012) |
| *AXAH5* | arabinoxylan arabinofuranosidase 5 | MLOC_72365 | 124 | GH51 | CAACTCATGGCAGCACTACGGATG | AGATGCAACCGTCTGATCGT | Laidlaw et al. (2012) |
| *Ara1* | α-l-arabinofuranosidase/β-d-xylosidase | MLOC_54544 | 177 | GH3 | CGTAGGGAGGATCGATTTCA | AACCGCAACAAAACAACTCC | Lee et al. (2003) |
| *Xyl* | β-d-xylosidase | MLOC_62475 | 106 | GH3 | AGCTGATCGGGTTCCAGAG | CGAAGCAGCAGGTTTATCGT | Lee et al. (2003) |
| *Cyclophilin* | cyclophilin | MLOC_4890 | 122 |  | CCTGTCGTGTCGTCGGTCTAAA | ACGCAGATCCAGCAGCCTAAAG | Vandesompele et al. (2002) |
| *GAPdH2-2* | glyceraldehyde-3-phosphate dehydrogenase | MLOC_18233 | 198 |  | GTGAGGCTGGTGCTGATTACG | TGGTGCAGCTAGCATTTGAGAC | Vandesompele et al. (2002) |
| *HSP70* | heat shock protein 70 | MLOC_12446 | 108 |  | CGACCAGGGCAACCGCACCAC | ACGGTGTTGATGGGGTTCATG | Vandesompele et al. (2002) |
| *Tubulin* | α-tubulin | MLOC_7079 | 248 |  | AGTGTCCTGTCCACCCACTC | AGCATGAAGTGGATCCTTGG | Vandesompele et al. (2002) |

**Additional references**

Banik, M., Garrett, T.P., and Fincher, G.B. (1996). Molecular cloning of cDNAs encoding (1→ 4)-β-xylan endohydrolases from the aleurone layer of germinated barley (*Hordeum vulgare*). *Plant Mol. Biol.* 31, 1163-1172.

Lee, R.C., Hrmova, M., Burton, R.A., Lahnstein, J., and Fincher, G.B. (2003). Bifunctional Family 3 Glycoside Hydrolases from Barley with α-l-Arabinofuranosidase and β-d-Xylosidase Activity characterization, primary structures, and cooh-terminal processing. *J. Biol. Chem.* 278, 5377-5387.

Slakeski, N., Baulcombe, D.C., Devos, K.M., Ahluwalia, B., Doan, D.N., and Fincher, G.B. (1990). Structure and tissue-specific regulation of genes encoding barley (1→ 3, 1→ 4)-β-glucan endohydrolases. *Mol. Gen. Genet.* 224, 437-449.
